# Revolutionizing cancer care strategies: immunotherapy, gene therapy, and molecular targeted therapy

**DOI:** 10.1007/s11033-023-09096-8

**Published:** 2024-01-28

**Authors:** Aasma Zafar, Muhammad Jawad Khan, Junaid Abu, Aisha Naeem

**Affiliations:** 1 Department of Biosciences, COMSATS University, Islamabad, 45550 Pakistan; 2 Hazm Mebaireek General Hospital, Hamad Medical Corporation, P.O. Box 3050, Doha, Qatar; 3 Qatar University Health, Qatar University, P.O. Box 2713, Doha, Qatar

**Keywords:** Immunotherapy, Gene therapy, Targeted molecular therapy

## Abstract

Despite the availability of technological advances in traditional anti-cancer therapies, there is a need for more precise and targeted cancer treatment strategies. The wide-ranging shortfalls of conventional anticancer therapies such as systematic toxicity, compromised life quality, and limited to severe side effects are major areas of concern of conventional cancer treatment approaches. Owing to the expansion of knowledge and technological advancements in the field of cancer biology, more innovative and safe anti-cancerous approaches such as immune therapy, gene therapy and targeted therapy are rapidly evolving with the aim to address the limitations of conventional therapies. The concept of immunotherapy began with the capability of coley toxins to stimulate toll-like receptors of immune cells to provoke an immune response against cancers. With an in-depth understating of the molecular mechanisms of carcinogenesis and their relationship to disease prognosis, molecular targeted therapy approaches, that inhibit or stimulate specific cancer-promoting or cancer-inhibitory molecules respectively, have offered promising outcomes. In this review, we evaluate the achievement and challenges of these technically advanced therapies with the aim of presenting the overall progress and perspective of each approach.

## Introduction

Cells acquire a cancerous phenotype due to a multitude of aberrant changes that manifest at the levels of proteins, RNA, or DNA. The year 2020 witnessed staggering statistics from the World Health Organization (WHO)—one-sixth of global deaths were attributed to cancer, underscoring the urgent necessity for safer, personalized, and more effective treatment modalities. While conventional anticancer methods such as surgery, radiotherapy, and hormonal therapy [1] have shown advancements, the realm of cancer therapeutics is abuzz with exploration aimed at enhancing survival rates. Within this landscape, emerging treatment avenues including immunotherapy, gene therapy, and molecular targeted therapy are offering promising prospects. These innovative therapeutic paradigms have historical roots, but it’s the availability of comprehensive genomic and individualized data that has truly refined their applications. The core objective of these groundbreaking treatments is to overcome the limitations inherent in traditional anticancer approaches—adverse treatment effects and long-term side effects.

**Fig. 1:**
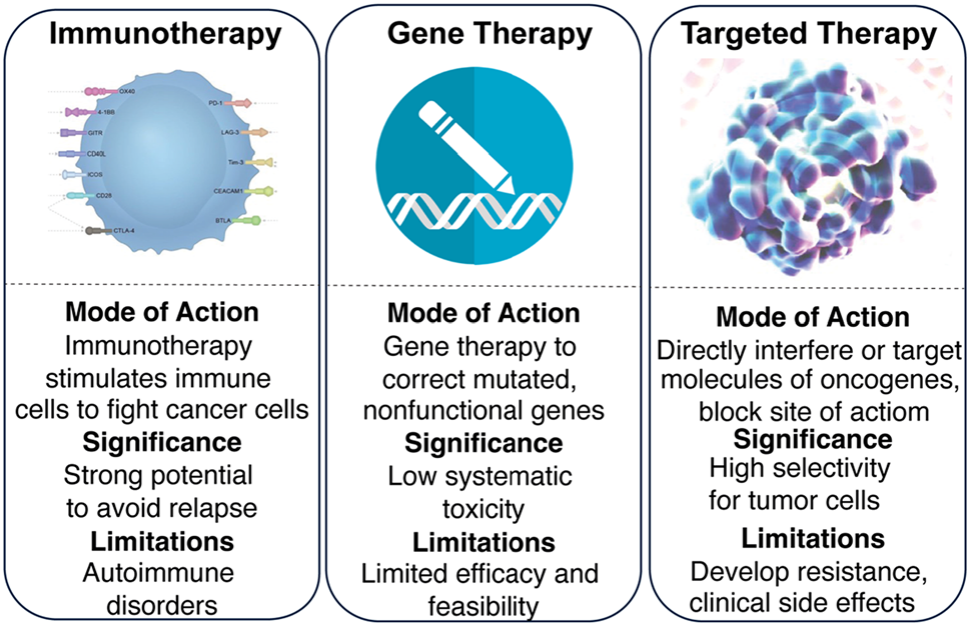
Abstract figure

In spite of these strides, cancer stands as the second leading cause of mortality, prompting an urgent quest for precise, targeted anticancer interventions to improve tolerance and mitigate both immediate and enduring side effects. The pursuit of better outcomes steers oncologists towards a strategy of integrated disease management, entailing dynamic treatment regimens that optimize cancer management. This article delves into anti-cancer therapeutic methods—immunotherapy, gene therapy, and molecular targeted therapy—tracing their historical development, assessing present progress, and outlining the potential they hold for the future (Fig. 1).

## Immunotherapy

The immunotherapy concept is based on directing one’s own immune response specifically towards cancerous cells [2]. With fewer off-targets compared to chemotherapy, immunotherapy magnetized its consideration for treating cancers. Various strategies have been ascertained to achieve activation or boosting of the immune system for fighting cancers.

### ***Immunostimulant***

**Fig. 2:**
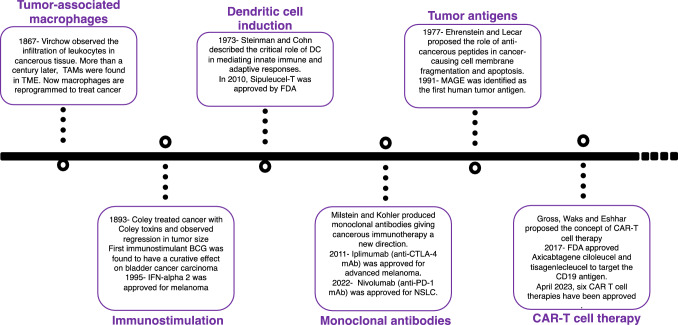
Histological milestones in immunotherapy: a chronological exploration of groundbreaking concepts

Immunostimulants work by exciting the immune system through various dynamics, such as the production of cytokines and specific antibodies, the release of α and γ interferons, and the activation of B and T lymphocytes [3]. In the early twentieth century, Coley observed a reduction in sarcoma size after injecting it with gram-positive bacteria. This observation led to the discovery of Coley toxin, a bacterial product that became the first known immunostimulant for cancers [4] (Fig. 2). However, its acceptance in the medical community and widespread use were encumbered by several reasons, such as inconsistent results and inadequately designed studies.

Later, in the 1960s, the recognition of the effectiveness of immunostimulants such as Bacillus Calmette–Guerin (BCG) in managing solid tumors, and its subsequent approval for some malignancies such as bladder cancer, sparked interest in exploring the potential of immunotherapy in treating cancer [5]. Subsequent studies on interferon-α (IFN-α), a cytokine, further demonstrated antitumoral activity in melanoma, hairy cell leukemia, renal cell carcinoma (RCC), and other solid tumors [6]. In 1986, IFN-α2 was approved to cure hairy cell leukemia, and it was developed as the first immunotherapy for adjuvant treatment of stage IIB/III melanoma in the USA in 1995 [7]. Soon after, another cytokine, interleukin-2 (IL-2), was approved by the FDA due to its anticancer activity in RCC and melanoma (Table 1). However, a high dosage of IL-2 was found to be associated with severe adversities, such as hemodynamic complications requiring hospitalization in an intensive care facility [8].

**Table 1:** List of chemotherapeutic agents for cancer treatment and their constraints

Types	Example	Applications	Risk factor	References
Immunotherapy				
Immunostimulation with lectin, IL-2, IL-5, IFN-α		Solid tumors	Hemodynamic complications	[6–8]
Peptide- and protein-based peptide vaccines		Various cancer types	Limited clinical significance at advanced stages	[11]
TLRL attached protein and peptide vaccines	Imiquimod, resiquimod	Skin cancer	Nausea, fever, muscle and joint pain, tiredness	[14, 15]
Heat shock protein-based vaccines	Hsp70.PC-F vaccine	Lung cancer		[19–21]
Monoclonal antibodies	Ipilimumab (anti-CTLA-4) Nivolumab Pembrolizumab Cemiplimab (anti-PD-1) Atezolimumab, Durvalumab and Avelumab (anti-PD-L) Bevacizumab Panitumumab Cetuximab Panitumumab Trastuzumab Rituximab	Melanoma, renal cell carcinoma, squamous cell carcinoma, and non-small cell lung cancer	Low efficacy and high toxicity for CTLA-4, Aggressive progression with PD1 Allergic reaction, arterial thromboembolic	[23–25]
		Colorectal cancer Head and neck, colorectal, breast cancer		[22, 28, 29, 31]
		Hodgkin lymphoma, non-hodgkin lymphoma, Chronic lymphocytic leukemia		[22, 28, 31]
Chimeric antigen receptor	Axicabtagene ciloleucel (axi-cel) Tisagenlecleucel (tisa-cel)	Large B cell lymphoma	Partial or complete loss of targeted antigen in recurrent cases, Cytokine-released syndrome and on-target off-tumor toxicities	[46, 47]
Gene therapy				
Oncolytic	G207 NV1020 TNFerade biologic Rexin-G *HSV-tk* TG01 Gendicine	Advanced pancreatic gliomas Liver cancer Locally advanced pancreatic cancer Many metastatic cancers Prostate cancer, Gliomas, Prostate cancer	Autoimmune response, infection	[52, 59, 60, 64–66, 71]
Targeted molecular therapy				
Tyrosine kinase inhibitor based anti-HER2 therapy	Lapatinib, Neratinib, Afatinib		Resistivity, may have skin, eyes, nails or hair problems	[77–80]
VEGF inhibitor	Bevacizumab	Colorectal, Breast, Renal cell carcinoma, Ovarian, glioma, NSCLC	Limited clinical significance for patients of advanced stages	[85, 86, 94]
PARP inhibitors	Olaparib, Niraparib, Talazoparib, Rucaparib	Cancers with mutated BRCA gene	Development of resistance	[88]
VEGFR inhibitors	Ramucirumab	Gastric cancer, Malignant hepatoma	Nausea, fever, muscle and joint pain, tiredness	[87]
EGFR inhibitors	Erlotinib, Afatinib, Gefitinib, Cetuximab	NSCLC, Pancreatic cancer, Colorectal cancer		[88–91, ]
ALK inhibitors	Crizotinib	NSCLC	Low efficacy and high toxicity for CTLA-4, Aggressive progression with PD-1 Allergic reaction, arterial thromboembolic	[86, 87]
mTOR inhibitors	Everolimus, Temsirolimus	Astrocytomas, renal adenocarcinoma		[84, 85]
CDK4/6 inhibitor	Palbociclib	Breast cancer	Autoimmune response, infection	[92, 93]
MDM2 inhibitor	Nutlin 3a	Solid malignances		[93]

**Fig. 3:**
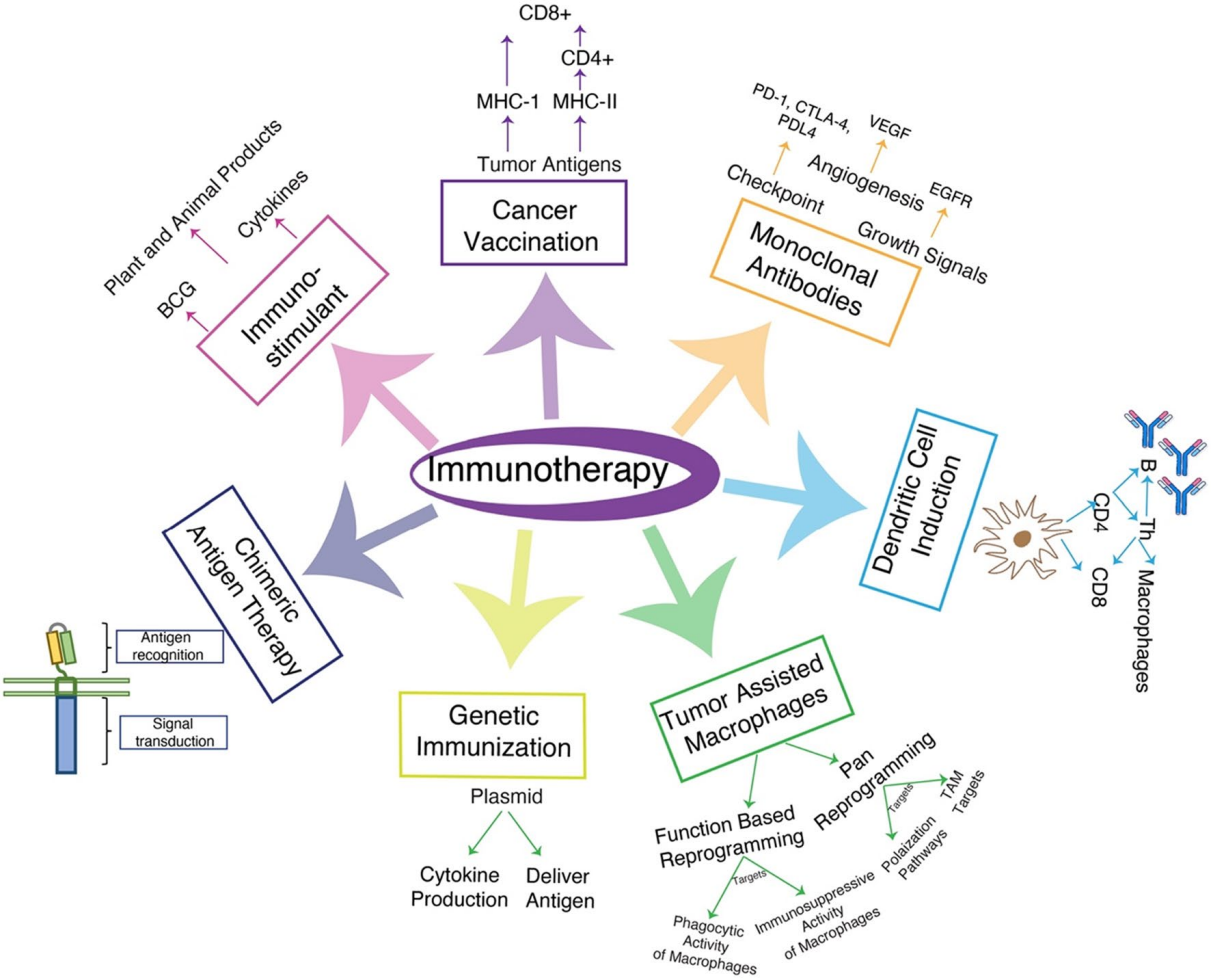
Different immunotherapeutic strategies. This figure illustrates diverse immunotherapeutic approaches for combating cancer: immunostimulants, certain bacterial, plant, or animal products enhance the immune response against cancer; cancer vaccination, stimulates the immune response by presenting tumor antigens through either MHC-I or MHC-II molecules; monoclonal antibodies, synthesized to target angiogenesis, checkpoint and growth factors, or deliver radioactive isotopes to cancerous cells; ex-vivo induction of dendritic cells, amplifies the immune response by mobilizing CD8+ T cells and macrophages to eliminate cancer cells and engineered dendritic cells also activate B cells for antibody production; reprogramming of macrophages, through pan programming and function-based programming, shifts macrophages from a pro-cancerous to an anti-cancerous role, genetic immunization strategy, introduces exogenous plasmids for cytokine production and antigen delivery to cancerous cells; chimeric antigen receptor (CAR) therapy, utilizes genetically modified immune cells expressing synthetic receptors that bind to tumor cells. *MHC-I* major histocompatibility molecule 1, *MHC-II* major histocompatibility molecule 2, *Th* helper T cells, *PD-1* programmed death-1, *PD-L* programmed death ligand, *CTLA-4* cytotoxic T-lymphocyte antigen-4

Bacterial products such as BCG, recombinant cytokines like interleukin and interferons, animal and plant-originated products, and synthetic drugs including levamisole, immunocynin, bestatin, and CpG oligonucleotides, as well as imiquimod, are being utilized for their immunostimulating function in cancer treatment [3] (Fig. 3).

### Cancer vaccination

The use of anti-tumor peptides as cancer vaccination emerged from the identification of various tumor antigens (TAs) that have the ability to stimulate T-cells against cancer [9]. Somatic mutations generate tumor-specific antigens, also known as neoantigens, while non-mutated but abnormal proteins due to misfolding, truncation, or abnormal post-translational modification are distinguished as tumor-associated protein [10]. These TAs stimulate cellular and/or humoral responses, giving rise to antigenic determinants presented as major histocompatibility complex (MHC) class I molecules at the surface of tumor cells to incite CD8+ T cells [11]. In addition, MHC class II fragments, either presented by antigen-presenting cells (APC) or tumor cells, are recognized and responded to by CD4+ T cells. However, being a fragment of MHC class I or class II, TAs can bind to specific MHC molecules and hold great clinical significance only for those who express that particular MHC molecule [9]. Some peptides from TAs can engage MHC class I and MHC class II molecules, building the foundation for the development of peptide- and protein-based vaccines for multiple cancer types [11] (Fig. 3).

A number of vaccines have been synthesized and tested for their effectiveness in different cancers. The limited clinical significance of first-generation vaccines was observed in a small group of patients in advanced stages of cancer [12]. However, it provided useful insight into the reactivity of tumor cells to counterbalance the immunization effects induced by protein and peptide vaccines.

To maximize specificity and effectiveness, many modifications have been proposed for peptide and protein-based vaccines, such as the utilization of immunological adjuvants that help the gradual discharge and consequent amplification of antigens to induce an immune response. Commonly used adjuvants include aluminum salts, oil-in-water emulsion (MF59), nontoxic derivatives from Salmonella (MPL), water-in-oil emulsions (Montanide ISA 51 and ISA 720), and the saponins (ISCOM, QS-21, AS01, and AS02) [13].

Another modification proposed in protein- and peptide-based vaccines is the insertion of toll-like receptor ligands (TLRL) such as TLR3L, TLR4L, TLR7/8L (imiquimod, resiquimod) and TLR9L (CpG), activating APCs [14]. Some TLRL, such as TLR3L, exhibited stimulatory potentials for APC and natural killer (NK) cells initiating tumor cell death [14]. TLR9L has been found to effectively stimulate the induction of TA-specified CD8+ T cells in advanced cancer patients [15]. Alternatively, granulocyte–macrophage colony-stimulating factor (GM-CSF) has been ascertained to weaken the vaccine-induced immune response for multi-peptide vaccines [16].

In 1986, gp96, an endoplasmic reticulum-residing member of HSP90 (heat shock proteins), was isolated from fibrosarcomas of mice after stimulation with methylcholanthrene A and was found to function as a tumor rejection antigen [17]. Extracellular HSP has been observed to play a stimulatory role for the immune system against tumorous tissue either by displaying immunogenic peptides originating from tumors or integrating innate and adaptive immunity through the secretion of chemokines, cytokines, and nitric oxide [18]. The gp96 and HSP70 peptide-based vaccines extracted from autologous tumor lysate were introduced in late-stage melanoma, metastatic colorectal cancer, glioma, and renal cell carcinoma patients [19].

Although HSP–peptide complex treatment incited the immune response in a majority of the patients, however, the response remained limited to certain patient subgroups. A clinical trial study with twelve brain tumor patients treated with recombinant HSP70 in an intra-tumoral manner after surgery exhibited complete clinical response (CR) along with the buildup of Th1 cell-mediated immune response and decline in immunosuppressive T_reg_ cell population [20]. Another clinical setting with twelve patients with non-resectable or reiterated HCV-related hepatocellular carcinoma advocates the effectiveness of the treatment [21].

### Monoclonal antibodies (mAbs)

Monoclonal antibody (mAbs) development represents a pivotal aspect of immunotherapeutic strategies aimed at suppressing the immunosuppressive influence of cancer cells [22] (Fig. 3). Köhler and Milstein [23] demonstrated the production of high-quantity mAbs displaying identical antigens in 1975. Since then various mAbs with diverse mechanisms of action have been developed, including those opposing neoplastic activity, neutralizing trophic signaling, or stimulating the immune system against tumor cells [24]. The initial investigation for the therapeutic role of mAbs failed most possibly due to the incompatibility of mice mAbs to recruit human immune effector molecules [22]. Initial investigations into the therapeutic role of mAbs faced challenges, possibly due to the incompatibility of mouse-derived mAbs with human immune effector molecules [22].

However, the development of chimeric or fully humanized mAbs has strengthened their efficacy, with studies validating their effectiveness in hematological and solid tumors [25]. The response rate to tumor-associated specific mAbs (e.g., trastuzumab) was observed at 35% in patients with advanced metastatic breast cancer when administered alone [25]. However, a marked increase in the survival rate, along with an improvement in the duration of response, was observed by combining trastuzumab with chemotherapy and/or radiotherapy [25].

Bevacizumab, a recombinant humanized mAb, binds vascular epithelial growth factor (VEGF) and inhibits angiogenesis [26]. It is approved for the treatment of colorectal cancer, glioblastoma (GB), non-squamous non-small cell lung cancer (NSCLC), and breast cancer [26]. Cetuximab, a recombinant chimeric mAb, targets EGFR, HER-1, and c-ErbB-1, preventing the binding of EGF with its ligand and promoting tumorigenesis [27]. Panitumumab, a recombinant human anti-EGFR mAb, competitively binds with EGFR, inhibiting the attachment of EGF and TGFα to the receptor [28]. Trastuzumab binds to the extracellular domain of EGFR-2 protein (HER-2) and is recommended for breast cancer patients [25]. Rituximab targets CD20 antigen of B lymphocytes, showing significance for non-Hodgkin lymphoma (NHL), Hodgkin lymphoma, and chronic lymphocytic leukemia (CLL) [22] (Table 1).

Immune checkpoint proteins, such as cytotoxic T-lymphocyte antigen-4 (CTLA-4) and programmed death (PD-1), play a crucial role in regulating T cell activation by balancing pro-inflammatory and anti-inflammatory signalling [29]. PD-1 has two ligands, PD-L1 and PD-L2, with PD-L1 expressed on both tumor and immune cells. When coupled with PD-1, it inhibits T cell multiplication and cytotoxicity [29]. Blocking these inhibitors with their antibodies resulted in satisfactory outcomes in in-vivo studies.

Since 2010, FDA-approved mAb drugs include Ipilimumab (anti-CTLA-4), Nivolumab, Pembrolizumab, and Cemiplimab (anti-PD-1), as well as Atezolimumab, Durvalumab, and Avelumab (anti PD-L). Ipilimumab and tremelimumab are mAb formulated to counteract the activity of CTLA-4 (molecule downregulating the activation of T cells through a homeostatic feedback loop) thus allowing the prolonged activation of T cells, restoration of proliferative potentials of T cells to enhance T-cell mediated immunity along with patient’s anti-tumor immune response [30]. However, clinical trial data indicate low efficacy and high toxicity in patients treated with anti-CTLA-4 [31] and resistance development in those treated with anti-PD-1 therapy [32] (Table 1). Mechanisms of non-responsiveness to immune checkpoint inhibitors include tumor mutational burden, PD-L expression, interferon signaling, and MHC-1 loss [29].

Ibritumomab and tositumomab radioconjugates deliver radioactive isotopes to intended cells [33]. Tositumomab radio-conjugate, a murine IgG2a-λ mAb, binds CD20 antigen expressed on B lymphocytes [33]. Iodine-131 tositumomab is a radio-iodized product of tositumomab covalently attached to iodine-131 [33]. Ibritumomab, an anti-CD20 mAb, is linked with the chelator tiuxetan, acting as a specific chelation site for yttrium-90 [33]. Alemtuzumab, that binds to CD52 and leads to cellular lysis [34]. It is recommended by the FDA for fludarabine-refractory CLL, with reported clinical significance for cutaneous T-cell lymphoma, peripheral T-cell lymphoma, and T-cell prolymphocytic leukemia.

Ipilimumab and tremelimumab are mAbs formulated to counteract CTLA-4 activity, allow prolonged activation of T cells, restore proliferative potentials and enhance T-cell mediated immunity [30].

With increased response rate and disease-free survival compared to chemotherapy, milder side effects generally caused by an allergic reaction due to the introduction of foreign proteins are observed [35]. However, the infrequent acute adverse effects such as arterial thromboembolic in patients treated with Bevacizumab- a mAb targeting VEGF [36] and autoimmune colitis caused by CTLA4 specific mAbs ipilimumab and tremelimumab [37] have been observed.

### Dendritic cell induction

Dendritic cells play a key role in mediating innate immunity and stimulating adaptive immunity (Fig. 3). The dysfunction of endogenous DCs in cancer patients has prompted the development of ex-vivo DCs with controlled loading of antigens, enhancing the specificity and magnitude of the T-cell response [38]. The ex-vivo generation of DCs allows the incorporation of supplementary features, such as tumor-relevant homing signals that direct the trafficking of immune cells toward potential metastatic sites. In-vivo DCs have the potential to acquire resistance to inhibitory factors like IL-10, TGF-β, VEGF, and IL-6 [38]. However, a notable increase in Tregs is observed in response to cancer vaccines, compromising the effectiveness of the vaccine [38].

Following its success in melanoma and follicular lymphoma, the clinical use of partially mature “first-generation” DCs has been explored in various tumor types [38]. However, the expression of costimulatory molecules and immunogens remained below the optimal level as compared to those arising from mature “second-generation” DCs. To address this, an improvement in the macrophage-conditioned medium and in the cytokine cocktail, including IL-1α, tumor necrosis factor-α (TNF-α), IL-6, and PGE2, was introduced to stimulate DCs and promote high expression of costimulatory molecules [38]. Compared to immature DCs, this cocktail exhibited enhanced immunogenic function along with an upgraded migratory response to lymph nodes [38].

### Genetic immunization of cancers

Various strategies have been proposed for genetically immunizing solid tumors, including cytokine gene therapy and plasmid-based immunization (Fig. 3). Previous attempts, such as injecting plasmid DNA encoding cytokines to stimulate an immune response against tumor cells, faced challenges due to a limited immunogenic response [39]. However, the plasmid-based immunization process, delivering antigens through viral and microbial vectors, has shown promising outcomes by eliciting both antibodies and cellular responses in mice [40].

Clinical trials evaluating the effectiveness of self-TAs, such as carcinoembryonic antigen (CEA) against colorectal cancer, confirmed the safety of DNA immunization [41]. Yet, the responsiveness to CEA varied among patients, with only four out of seventeen showing an immune response, highlighting the inadequacy of plasmid DNA immunization in stimulating a T-cell response [41]. Similarly, a study involving MART-1 plasmid injection intramuscularly in melanoma patients reported no increase in immunity [42]. The challenge lies in achieving a balanced response between neutralizing antibodies and the expanding population of Treg cells to self-antigens, hindering the improvement of immunity against cancer [43]. A study utilizing an alpha-virus plasmid carrying the CEA antigen gene enveloped in virus-like replicon particles (VRP) claimed a reduction in the neutralization effect caused by antibodies and Treg cells, leading to an improvement in immunotherapeutic treatment and overall survival rate [43].

### Tumor-associated macrophages

Tumor-associated macrophages (TAMs) are immune cells abundantly present in the tumor microenvironment (TME) [44]. They play a dual role, acting as tumor inhibitors during initiation stages and as tumor promoters in advanced stages [44]. The presence of macrophages contributes synergistically to therapeutic responses, such as increased sensitization to 5-FU adjuvant therapy [44]. Although strategies to inactivate or deplete macrophages have been employed, these attempts were unsuccessful due to an attenuated immune response and significant repression of intra-tumor neutrophils [44]. Consequently, the idea of readjusting macrophages from a pro-tumor to an anti-tumor state gained momentum.

Two categories of macrophage reprogramming include pan-reprogramming and function-based reprogramming (Fig. 3) [44]. Pan-reprogramming targets signaling pathways aiding polarization to a pro-tumor state or those preferentially expressed in TAMs. Histone deacetylases (HDACs), phosphoinositide 3-kinase gamma (PI3Kγ), leukocyte immunoglobulin-like receptors B-2 (LILRB-2), and macrophage receptors with a collagenous structure (MARCO) are utilized in this approach [44]. On the other hand, function-based reprogramming targets specific roles of macrophages such as immunosuppression and phagocytosis [44]. The function-based reprogramming strategy targets the tumor-macrophage axis, such as the antiphagocytic signal CD47-SIRP1α, β2-M-LILRB1, and CD24-SIGLEC-10, proving effective in various cancers [44].

### Chimeric antigen receptor therapy (CAR therapy)

Chimeric antigen receptor T (CAR-T) cell therapy is gaining widespread acceptance due to its durable and effective CRs. Engineered synthetic receptors direct lymphocytes, typically T cells, to recognize and eradicate cells expressing specific antigens (Fig. 3). Since 2017, various CAR-T products have received approval from the FDA. Axicabtagene ciloleucel (axi-cel) and tisagenlecleucel (tisa-cel) target the CD19 antigen in patients with large B-cell Lymphoma (Table 1). However, antigen escape, a phenomenon observed in patients treated with single antigen-targeting CAR-T, results in complete or partial loss of that antigen in 30% of relapsed/recurrence cases [45]. B cell maturation antigen (BCMA) and CD38 are identified as target antigens for multiple myelomas. Treating relapsed and refractory multiple myeloma patients with BCMA-CD38-CAR-T therapy yielded a high response rate and low recurrence rate [46]. Clinical trials for various solid malignancies, including GB, renal cell carcinoma, lung cancer, and hepatocarcinoma, are ongoing.

Second, third, and fourth-generation CAR-T cells are not only generated but are undergoing continuous refinement and enhancement. These iterations of CAR-T cell therapies represent progressive advancements in the design and functionality of CAR-T cells. Second-generation CAR-T cells were characterized by the addition of one co-stimulatory domain to the original CAR structure, improving their efficacy. Third-generation CAR-T cells incorporated multiple co-stimulatory domains, further enhancing their activation and persistence within the TME. The fourth generation introduces additional features, such as cytokine secretion or genetic modifications, aiming to address challenges like antigen escape and the hostile conditions of the TME. These advancements signify a dynamic field of research and development, continually pushing the boundaries of CAR-T cell therapy for improved outcomes in cancer treatment.

Despite attempts to stimulate the immune system through various approaches, challenges in the form of generating autoimmune disorders exist. While CAR-T displays promising outcomes for hematological malignancies, its effectiveness in solid tumors remains compromised. Cytokine-release syndrome and on-target off-tumor toxicities are major challenges that need to be resolved for effective CAR-T therapy. Current research is focused on improving immunotherapy and addressing the issues of autoimmune disorders. Moreover, with successful immunotherapy, the chances of cancer recurrence will diminish, making the dream of cancer-free survival a reality. Furthermore, the high cost and labor-intensive process involved in generating CAR-T cells render it inaccessible to many patients.

## Gene therapy

Gene therapy holds great promise for treating cancer, employing various approaches that stimulate immune responses against cancer cells, utilize oncolytic viruses to kill cancerous cells, and suppress cancer survival and supportive activities. This article will discuss these strategies in detail.

### Genetic modulation of the immune system

A phase I study investigating the safety and efficacy of poxviral vaccine-based treatment containing genes for CEA and MUC-1 together with TRICOM (a triad of co-stimulatory molecules) comprising B7-1, ICAM-u1, and LFA-3 engineered in vaccinia and fowl pox determined the increased endured response by the immune system in patients with ovarian cancer and breast cancer [47].

Numerous attempts to manipulate the immune response have been reported in the literature [48]. Strengthening the immune response involves directing immune cells against cancer cells through the enhanced production of proinflammatory, immune-stimulating molecules initiated by incorporating one or more genes into cancer cells [48]. Transfusion of mononuclear circulating blood cells modified with an immunostimulatory gene or TA into the patient’s body triggers an immune system response targeting cancer cells [49]. For instance, a study involving TG01 (the first immunotherapy drug targeting KRAS mutations) along with GM-CSF and gemcitabine in an adjuvant setting for patients with resected pancreatic adenocarcinoma demonstrated increased activation of the immune response, extension in overall survival, and disease-free survival rate with improved tolerance [50]. A Phase I study assessed the safety and efficacy of a poxviral vaccine-based treatment. This treatment included genes for CEA and MUC-1, along with TRICOM (a triad of co-stimulatory molecules: B7-1, ICAM-u1, and LFA-3) engineered in vaccinia and fowlpox. The study determined an enhanced and sustained immune response in patients with ovarian cancer and breast cancer [47].

### Oncolytic gene therapy

Oncolytic gene therapy entails the introduction of genetically modified viruses into the body to eliminate cancerous cells, either through the expression of cytotoxic proteins or cytolysis induced by the virus’s propagation (Fig. 3). Viruses such as vaccinia, adenovirus, herpes simplex virus type I (HSV-1), reovirus, and Newcastle disease virus are chosen for their inherent ability to infect cancer cells or due to their easy genetic manipulation [51]. In various animal models, including murine and canine studies, a noteworthy increase in the survival rate coupled with a reduction in metastasis has been reported, demonstrating the potential of oncolytic gene therapy [52]. An overview of oncolytic gene therapy agents, either FDA-approved or under current investigation, is provided in Table 1.

The oncolytic adenovirus (OAd) Delta-24-RGDOX, expressing OX40L (an immune co-stimulator) alongside an anti-PD-L1 antibody in glioma-bearing mice, significantly prolonged the survival rate to 85%, compared to the control survival rate of 28% [53]. Utilizing a humanized monoclonal CTLA-4 antibody expressing oncolytic adenovirus Ad5/3-Δ24aCTLA4 in peripheral blood mononuclear cells (PBMC) of four cancer patients in advanced stages achieved increased activation of T cells, evidenced by a rise in IL-2 [54]. A recent study featuring rAd.sT (telomerase reverse transcriptase promoter-regulated oncolytic adenovirus) combined with a soluble transforming growth factor receptor II and human IgG Fc fragment (sTGFβRIIFc) gene demonstrated dose-dependent cytotoxicity in breast and kidney cancer patients [55]. Furthermore, the intratumoral introduction of rAd.sT in the immunocompetent breast cancer mice model impeded tumor progression and metastasis in lungs and liver synergistically with anti-CTLA-4 and anti-PD-1 antibodies [55].

The modeling of HSV-1 positions it as an effective and selective eradicator of cancerous cells. A recent study focusing on metastatically advanced pediatric glioma revealed the efficacy of G207 (genetically modified HSV-1), either alone or accompanied by radiation with manageable consequences [56]. In a phase I/II investigation with NV1020 (recombinant HSV-1) on colorectal cancer patients experiencing high-grade metastasized disease, it exhibited potential for stabilizing hepatic metastasis and intensifying responsiveness to cancers by enhancing the sensitivity of tumorous cells to chemotherapy and inciting a tumor-lytic immune response [57]. Enhanced controlled cytotoxicity is achieved by equipping oncolytic (HSV-1) with a bi-specific T cell engager (BiTE) connecting PD-L1 (expressed on tumor cells) and CD3ε (expressed on T cells) in malignant hydroperitoneum derived from patients with different cancers [58].

### Gene transfer strategic approach

Gene transfer offers another avenue for cancer treatment by facilitating the introduction of foreign genes into either cancerous cells or surrounding tissues. Suicide genes, anti-angiogenesis genes, and genes promoting cellular stasis have been proposed as favorable choices for impeding cancer progression [59]. Various methods including viral transfer, naked DNA transfer, oligodendromer DNA coating and electroporation are recognized as practical [59]. However, the chosen delivery method influences expression duration and specifications for gene transfer therapy. For example, adenoviral vectors are commonly selected to deliver HSVtk gene for transient expression required to induce cellular mortality. In contrast, to counteract angiogenesis, prolonged expression of sFLT-1 and statin-AE genes is essential. Therefore, their delivery is carried out through plasmids containing transposons for gene insertion into cellular DNA [60]. A clinical study involving TNFerade biologic (adenovector delivering tumor necrosis factor-alpha (TNF-α) to tumor cells) in locally advanced pancreatic cancer patients confirmed its safety but failed to demonstrate an extension in survival [61].

Rexin-G is a gene therapy with a broad spectrum of tumoricidal activity designed to target lesions by binding to unusual signature (SIG) proteins in the tumorous microenvironment. It encodes a cytocidal dominant-negative mutant construct of cyclin G1 (dnG1) in tumorous cells, disrupting the activity of wild-type cyclin G1, eventually causing cell growth arrest [62].

Another gene transfer approach involves the introduction of HSV-tk followed by the administration of ganciclovir (GCV), an anti-herpetic prodrug that exhibits no toxicity for human cells unless phosphorylated by HSVtk [63]. A Phase 1 study with locally relapsed prostate cancer established its safety profile along with anti-tumorigenic activity [64]. Furthermore, an unsystematic study on high-grade glioma revealed an 81% improvement in survival rates with no serious health hazards [65].

Approximately 50% of cancers carry mutated p53 genes to evade apoptosis apoptosis [66]. INGN-201, an adenovector, was developed to deliver p53 to cancerous cells. Clinical testing of INGN-201 in prostate cancer exhibited a high safety profile with increased expression of p53 in cancerous tissue, compelling them to undergo apoptosis [64]. Additionally, the efficacy of Ad-p53 has been noted NSCLC with a significant regression of the disease either given singly or in combination with radiation and chemotherapy [67]. Gendicine, a recombinant adenovirus transferring p53, accompanied by chemotherapy, enhanced therapeutic significance while reducing the harmful consequences of chemotherapeutic agents in head and neck cancer patients [68].

## Molecular targeted therapy

Targeting molecules crucial for cancer progression and survival allows for specific cancer treatment. Numerous molecular targets have undergone clinical assessment for their anti-cancer potential, leading to the approval of various molecular therapies by FDA. These therapies have demonstrated remarkable success in treating diverse cancers, including breast, lung, ovarian, leukemia, and colorectal cancers [69].

### Inducing apoptosis through molecular targeting

To induce apoptosis in cancerous tissues, several molecules have been identified as potential targets. HER2 is critical in 15–20% of breast cancers, regulating key cell proliferation pathways such as mitogen-activated protein kinase (MAPK) and phosphatidylinositol 3-kinase (PI3K)–AKT [70]. Molecular therapies targeting HER2 include mAbs (Trastuzumab and Pertuzumab), an antibody–drug conjugate (T-DM1), and small molecule tyrosine kinase inhibitors (Lapatinib, Neratinib, and Afatinib) are approved by FDA (Table 1). Co-administration of trastuzumab with chemotherapeutic drugs in an adjuvant setting improves the survival rates [71].

Phosphoinositide 3-kinases alpha and delta (PI3Kα, PI3δ) play a crucial role in regulating cell death and growth. Idelalisib, a PI3Kδ inhibitor, was approved by the FDA in 2004 to treat CLL patients. Copanlisib, a co-inhibitor for PI3Kα and PI3δ, received FDA approval in 2017 for patients with follicular lymphoma [72]. Inhibiting AKT is achieved with MK-2206 (an AKT inhibitor). Everolimus and Temsirolimus, mTOR inhibitors, have shown efficacy in treating specific cancers [73, 74].

Crizotinib, a kinase inhibitor, is approved for NSCLC patients with ALK and ROS1 positive tumors. It has demonstrated increased survival rates in ALK-positive progressive NSCLC [75]. Inhibiting Poly (ADP-ribose) polymerases (PARP) is significant in treating cancers carrying BRCA mutations. Olaparib, Niraparib, Talazoparib, and Rucaparib are developed for inhibiting PARP in cancers with mutated BRCA gene [76] (Table 1).

Our recent findings highlight the atypical involvement of PI3K-Akt kinases and p53 in triggering both cell death and resistance, facilitated by the FDA-approved thymosin beta 4 (Tβ4) drug in medulloblastoma (MB) cells [77, 78]. The success of these groundbreaking studies, unraveling Tβ4’s influence on MB and GB cells, sets the stage for future investigations into the intricate roles of p53 and PI3K/AKT in cancer cells characterized by elevated p53 levels [79]. This research is poised to guide the development of methodologies assessing p53 levels in patient specimens and potentially pave the way for the clinical application of Tβ4.

B cell lymphoma 2 (BCL2) is a critical regulator of cell death. The inhibitors for BCL2 have been tested for their clinical significance in NHL, chronic lymphoid leukemia (CLL) and acute myeloid lymphoma (AML). In 2016, FDA approved Venetoclax for CLL patients having a deletion of the shorter arm of chromosome 17. Co-inhibitors for BCL2 and BCLX including ABT-737 and ABT-263 have been developed [80]. ABT-737 monotherapy is effective in suppressing tumor progression in NSCLC and lymphomas [81]. Clinical trials with ABT-263 yielded a response in 34.6% of patients while its combination with Rituximab significantly improved the response rate [82].

Inhibiting murine double minute (MDM2), an inhibitor of TP53, is of particular interest in developing anti-cancer therapies. Molecules like Nutlin 3a, RG7112, RG7388, AMG-232, APG-115, BI-907828, CGM097, DS-3032, and HDM201 have been synthesized to disrupt the MDM2-TP53 regulatory loop, inducing death in cancer cells [83] (Table 1). These targeted therapies showcase promising avenues for precise cancer treatment.

### Targeting key molecules of angiogenesis

Targeting angiogenesis to deprive cancerous cells of essential nutritional resources has emerged as an effective strategy for eradicating cancer. Bevacizumab, the first humanized mAB, binds to the circulating VEGF-A isoform, preventing its interaction with VEGFR and inhibiting the activation of VEGF signaling crucial for neovascularization. Combining bevacizumab with chemotherapy has shown promising outcomes in treating various solid tumors, including colorectal cancer [84], NSCLC [84], breast cancer [84], renal cell adenocarcinoma [84], ovarian tumor [85] and GB [86]. Another humanized mAB, Ramucirumab, engages VEGFR, leading to the inhibition of neovascularization. Clinical trials such as REACH for malignant hepatoma, RAINBOW, and REGARD for gastric cancer demonstrated encouraging outcomes when combining Ramucirumab with chemotherapeutic agents [87].

Inhibitors targeting biomarkers overexpressed in cancer cells have gained attention, particularly EGFR. EGFR inhibitors like Erlotinib, Afatinib, Gefitinib, and Cetuximab are commonly used (Table 1). A study administering Erlotinib to NSCLC patients previously treated with chemotherapy revealed a survival advantage with a significant improvement in the quality of life [88]. Pancreatic carcinoma patients treated with Erlotinib plus gemcitabine showed clinically favorable outcomes compared to gemcitabine alone [89]. The LUX-Lung 5 trial demonstrated improved outcomes when patients with resistance to Erlotinib or Gefitinib were treated with Afatinib and Paclitaxel [90]. Cetuximab has been observed to enhance survival and quality of life in colorectal cancer patients unresponsive to other therapeutic options [91] (Table 1). Targeting these key molecules involved in angiogenesis holds promise for effective cancer treatment.

### Targeting proliferation

An exciting anticancer strategy involves impeding the progression of the cell cycle through the inhibition of cell cycle regulatory proteins. CDK inhibitors, including Palbociclib, Ribociclib, and Abemaciclib, have been developed and are being evaluated for their clinical potentials [92]. For instance, Palbociclib, an anti-CDK4/6 inhibitor, when combined with letrozole (a hormone-based therapeutic agent), demonstrates a delay in disease progression beyond what is achieved with letrozole alone in ER+HER2 metastatic breast cancer (Table 1). However, it’s essential to note that the combination treatment does come with pronounced myelosuppression [93]. Nevertheless, the FDA has approved Palbociclib for treating HER2 and HR-positive metastasized breast cancer in combination with hormonal therapy, highlighting its clinical significance [92].

In a similar manner, inhibitors for WEE, such as AZD1775 and ZN-c3, have been developed and are currently under evaluation in clinical trials [94]. While molecular targeted therapy has facilitated the personalization of medicine, the persistent challenge of drug resistance remains. Additionally, it can lead to clonal selection, allowing other molecular subtypes to flourish, ultimately resulting in the rapid progression of cancer. Addressing these challenges is crucial for ensuring the long-term efficacy of targeted proliferation therapies.

## Conclusion

Until now multiple therapeutic strategies have been devised to treat cancer. While conventional therapies have served as a cornerstone in cancer management, their limitations, such as the development of treatment resistance and tumor relapse, have paved the way for advanced therapeutic approaches. These innovative strategies aim to address the challenges posed by traditional treatments. Recognizing the pivotal role of the immune system in cancer progression, scientists have explored the modulation of immune cells to achieve targeted immune responses against circulating cancer cells. The inherent heterogeneity of cancer cells and the concept of personalized medicine have further propelled the exploration of gene and targeted molecular therapies in cancer treatment. The integration of advanced therapies has not only enhanced the ability of clinicians to manage cancer effectively but has also provided researchers with the opportunity to refine these approaches for optimal anti-cancer solutions. Despite inherent shortcomings in each proposed solution, the current management of cancer involves the judicious utilization of both traditional and advanced approaches to treat various forms of cancer. The inclusion of advanced therapies has shown significant improvements in cancer management, leading to enhanced survival rates. However, the ultimate goal of achieving disease-free survival for all cancer patients, irrespective of tumor grade and stage, remains a formidable challenge yet to be fully realized.

## Data Availability

Not applicable.
